# *Sirt1* Promotes Cardiomyocyte Differentiation Through the XR_951230.1/miR-3663-3p/SMYD1 Axis

**DOI:** 10.3390/genes17030282

**Published:** 2026-02-27

**Authors:** Chengyu Li, Mairepati Mahemuti, Yusupujiang Maimaiti, Ting Wang, Xin Zhang, Zeyidan Jiapaer

**Affiliations:** 1 Xinjiang Key Laboratory of Biological Resources and Genetic Engineering, College of Life Science & Technology, Xinjiang University, Urumqi 830046, China; lichengyu970524@163.com (C.L.); m2521339985@163.com (M.M.); yusup_mmt@163.com (Y.M.); ting_w0208@163.com (T.W.); 2 School of Life Science and Technology, ShanghaiTech University, Shanghai 200031, China; 3 Tongji Hospital, School of Medicine, Tongji University, Shanghai 200092, China

**Keywords:** *Sirt 1*, cardiomyocytes, human embryonic stem cells, ceRNA network

## Abstract

**Background:** Sirtuin 1 (*Sirt1*) is known to regulate stem cell differentiation and cardiomyocyte function, yet its specific role and mechanism in human embryonic stem cell (hESC) differentiation into cardiomyocytes remain unclear. This study aimed to elucidate the functional contribution and molecular pathway of *Sirt1* in cardiomyogenesis. **Methods**: A *Sirt1* knockout (*Sirt1*^−^/^−^) hESC line was generated using CRISPR-Cas9 technology. The expression of key differentiation markers was analyzed by RT-qPCR at days 6, 8, and 9. The underlying mechanism was investigated through integrated RNA-sequencing (RNA-seq) analysis and dual-luciferase reporter assays. **Results**: *Sirt1* deletion significantly downregulated the expression of mesodermal (TBX6, KDR), cardiac precursor (NKX2.5, TBX5), and mature cardiomyocyte (cTNT, Hand2) markers. Mechanistically, a competing endogenous RNA (ceRNA) axis, LncRNA XR_951230.1/miR-3663-3p/SMYD1, was identified. *Sirt1* knockout reduced XR_951230.1 expression, which consequently elevated miR-3663-3p activity and suppressed its target gene SMYD1. **Conclusions**: These findings indicate that *Sirt1* is essential for promoting hESC differentiation into cardiomyocytes, potentially via the XR_951230.1/miR-3663-3p/SMYD1 pathway. This study provides new insights into the regulatory network of stem cell-based cardiomyogenesis and suggests potential targets for stem cell-based cardiac disease therapy.

## 1. Introduction

Sirtuin 1 (*Sirt1*) is a highly conserved member of the nicotinamide adenine dinucleotide (NAD+)-dependent Sirtuin family, which plays crucial roles in various biological processes, including inflammation, oxidative stress response, metabolism homeostasis, apoptosis, cell proliferation, migration, and invasion [1]. Recent advances in *Sirt1* highlight its critical regulatory roles in stem cell differentiation, embryonic development, neurogenesis, and chondrogenesis. Notably, accumulating evidence links *Sirt1* deficiency to severe cardiac developmental defects. Cheng et al. demonstrated that *Sirt1* is highly expressed in the mammalian heart, and its knockout in mice results in embryonic lethality accompanied by cardiac malformations, including ventricular septal defects and atrial septal defects [2]. According to Yi et al., *Sirt1* knockout in mice inhibits the expression of chemotactic genes (CXCL12/CXCR4 and CCL2/CCR2/CCR4) during cardiomyocyte maturation. causing perinatal cardiomyocyte and myofibril disorganization in vivo and impairing cardiac morphogenesis and development [3]. Additionally, *Sirt1*-deficient mice exhibited dilated cardiomyopathy, a phenotype closely linked to mitochondrial dysfunction [4]. Although *Sirt1* has been established as an indispensable cardioprotective regulator during embryonic cardiac development, the precise epigenetic mechanisms through which *Sirt1* modulates post-transcriptional events to govern human embryonic stem cell (hESC)-derived cardiomyocyte differentiation remain poorly defined.

Cardiomyocytes (CMs) are terminally differentiated cells that originate from the mesoderm during early embryogenesis, constituting the majority of cardiac tissue [5]. Cardiomyocyte differentiation is a highly orchestrated process tightly regulated by a network of core transcription factors (e.g., Mesp1, MEF2C, GATA4, TBX5, and NKx2.5) and conserved signaling pathways, including Wnt, FGF, and BMP cascades [6]. However, the limited proliferative capacity of primary cardiomyocytes and inherent species-specific differences in animal models pose significant challenges to studying human cardiac development. Human embryonic stem cells (hESCs) accordingly offer an ideal in vitro model to dissect the molecular circuitry underlying human cardiomyocyte differentiation, owing to their pluripotency and capacity for directed lineage commitment. Indeed, numerous studies have validated that hESCs possess a robust capacity to differentiate into diverse terminally differentiated cell types in vitro, including neurons, chondrocytes, and hepatocytes [7,8,9,10], making them ideal for dissecting the human-specific regulatory role of *Sirt1* in early cardiomyocyte differentiation.

Non-coding RNAs (ncRNAs), particularly microRNAs (miRNAs) such as miR-1, miR-133 [11], and miR-218 [12], serve as key regulators of cardiac development and cardiomyocyte maturation. During this process, miRNAs assemble with Argonaute (Ago) family proteins to form RNA-induced silencing complexes (RISCs), which recognize specific target mRNAs via base-pair complementarity and mediate post-transcriptional silencing of gene expression [13]. Long non-coding RNAs (lncRNAs), which encompass thousands of transcriptional loci in mammals, regulate diverse biological processes primarily through modulating transcription, post-transcriptional modification, and mRNA splicing [14,15]. Notably, lncRNAs can function as competitive endogenous RNAs (ceRNAs) that sequester miRNAs, thereby alleviating miRNA-mediated repression of downstream target mRNAs and fine-tuning gene expression networks [16]. Wu Yukang et al. demonstrated that elevated lncRNA-Cmarr in exosomes acts as a ceRNA by targeting miR-540-3p, thereby relieving the suppressive effect of miR-540-3p on Dtna expression and promoting the phenotypic maturation of embryonic stem cell-derived cardiomyocytes [17]. Notably, emerging evidence links *Sirt1* to ncRNA-mediated gene regulation in other developmental processes [18,19], but its role in ceRNA networks during early human cardiomyogenesis remains unexplored.

In this study, we generated a *Sirt1*-knockout human embryonic stem cell (hESC) model via CRISPR-Cas9, systematically uncovering the functional role of *Sirt1* in cardiomyogenesis. Our findings demonstrate that *Sirt1* knockout (*Sirt1*^−^/^−^) significantly inhibits the early stage of hESC cardiomyocyte differentiation, with mechanistic investigations identifying the LncRNA XR_951230.1/miR-3663-3p/SMYD1 axis as a key mediator. This work not only uncovers a novel *Sirt1*-dependent ceRNA pathway for early cardiac differentiation but also provides a precise molecular target for hESC-derived cardiomyocyte modulation, shedding light on congenital heart disease pathogenesis.

## 2. Materials and Methods

### 2.1. Cell Culture and Differentiation

The hESC line H9 was cultured in Essential 8™ medium (DMEM) (Gibco, Frederick, MD, USA) under humidified conditions at 37 °C and 5% CO_2_ [20]. for induction of cardiomyocyte differentiation, hESCs were dissociated into single cells using Versene (Invitrogen, Carlsbad, CA, USA) and seeded at a density of 4 × 10^5^ cells per well in Matrigel-coated 12-well plates using Essential 8™ medium supplemented with Y27632. On day 3 of differentiation, aspirate the culture medium and add 1 mL/well of RPMI 1640 Medium (Thermo Fisher Scientific, Waltham, MA, USA) supplemented with B-27 Supplement minus insulin (Thermo Fisher Scientific, Waltham, MA, USA) (RPMI/B27-insulin) and containing 5 μM IWP-4 for 48 h of incubation. On day 5, replace the culture medium with fresh RPMI/B27 (−insulin) medium for another 48 h; On day 7, replace the culture medium with 1 mL/well of RPMI/B27 (+Insulin) medium, followed by continuous culture for 72 h; On day 10, cells were subjected to starvation culture using glucose-free DMEM supplemented with 4 mM sodium L-lactate to enhance cardiomyocyte maturation. On day 13, replace the culture medium with glucose-free DMEM containing 4 mM sodium L-lactate for an additional 48 h; and from day 15 onwards, the starvation medium was switched back to RBI medium, which was refreshed every 72 h thereafter to maintain cell viability and maturation [21].

### 2.2. Sirt1 Knockout in hESCs

A small guide RNA (sgRNA) targeting the human *Sirt1* gene was designed using the CRISPR DESIGN online tool (http://crispor.org (accessed on 25 September 2015)), with selection based on high-scoring sequences and low off-target risk. The sgRNA sequence was commercially synthesized; corresponding primer sequences are detailed in Table S1. The constructed *Sirt1* knockout (KO) sgRNA expression plasmid was introduced into human embryonic stem cells (hESCs) via electroporation. Twenty-four hours post-transfection, a pool of successfully transfected cells was enriched by a 7-day puromycin selection. Single-cell clones were subsequently isolated using the limited dilution method and expanded in culture. *Sirt1* knockout efficiency was validated by Western blot analysis, ultimately yielding a *Sirt1* knockout monoclonal cell line. The knockout cells and their corresponding control cells used in the experiment were seeded onto Matrigel-coated culture plates. Within 24–48 h post-seeding, Essential 8™ complete medium supplemented with 10 μM Y-27632 was added, followed by replacement with standard Essential 8™ complete medium. Cultures were maintained at 37 °C in a 5% CO_2_ incubator.

### 2.3. Dual-Luciferase Reporter Assay

Cells were co-transfected with the constructed reporter plasmids (XR_951230.1-WT/Mut or SMYD1-WT/Mut) and either hsa-miR-3663-3p mimetic or negative control mimetic (mimetic-NC). At 48 h post-transfection, luciferase activities (firefly and Renilla) were measured using a dual-luciferase reporter assay kit (Promega, Madison, WI, USA) according to the manufacturer’s instructions [22]. The detailed sequences of the WT and Mut fragments inserted into the reporter plasmids are listed in Table S2.

### 2.4. RNA Extraction

Total RNA was isolated from hESC-derived cardiomyocytes using TRIzol reagent (BIOsharp, Anhui, China) in accordance with the manufacturer’s protocol. The concentration and purity of the extracted total RNA were determined using a NanoDrop 2000 spectrophotometer (Thermo Fisher Scientific, Waltham, MA, USA). For the qRT-PCR assay, cDNA was synthesized using the HiScript III 1st Strand cDNA Synthesis Kit (Vazyme, Jiangsu, China). Relative expression levels of target genes were determined using TB Green^®^ Premix Ex Taq™ II (TakaRa, Kyoto, Japan). Real-time PCR was performed using the ABI (Foster City, CA, USA) StepOnePlus Real-Time PCR System. GAPDH was used as the internal reference gene for mRNAs, while U6 small nuclear RNA served as the reference for miRNAs. Relative expression levels of target genes were calculated using the 2^−ΔΔCt^ method. All primer sequences used in this study are listed in Table S3.

### 2.5. Western Blot

Total cellular proteins were extracted using RIPA lysis buffer (1:100 dilution) supplemented with a protease inhibitor cocktail. Protein concentration was quantified via the BCA Protein Assay Kit (Thermo Fisher Scientific, Waltham, MA, USA) and normalized to ensure equal loading. Equal amounts of total proteins were separated by sodium dodecyl sulfate–polyacrylamide gel electrophoresis (SDS-PAGE) and electrophoretically transferred onto polyvinylidene fluoride (PVDF) membranes (Sigma-Aldrich, St. Louis, MO, USA). The membrane was blocked with 5% nonfat dry milk (1 h at room temperature), followed by overnight incubation at 4 °C with *Sirt1* monoclonal antibody (1:1000 dilution, SANTA, Redmond, WA, USA) and anti-GAPDH antibody (1:1000 dilution, Proteintech, Rosemont, IL, USA). The membrane was incubated with a secondary antibody (1:1000 dilution) at room temperature for 1 h, followed by another wash with PBST. Finally, protein bands were visualized using an ECL chemiluminescence detection kit (Biosharp, Hefei, China) and imaged with a Bio-Rad ChemiDoc™ Imaging System (Bio-Rad Laboratories, Hercules, CA, USA).

### 2.6. RNA-Sequencing

Collected cell samples on day 6 (*Sirt1*^−^/^−^ and WT). Total RNA was extracted using RNAiso Plus (Takara Bio, Kyoto, Japan). RNA quality and concentration were assessed using a NanoDrop 2000 spectrophotometer (Thermo Fisher Scientific, Waltham, MA, USA) and Agilent 2100 Bioanalyzer (Agilent Technologies, Santa Clara, CA, USA). Samples with A260/A280 ratios consistently maintained between 1.9 and 2.0 were selected for subsequent sequencing analysis. Purified total RNA underwent sequential 3′ and 5′ sequencing adapter ligation, reverse transcription, and PCR amplification. Libraries passing quality control were sequenced on the Illumina NovaSeq platform.

### 2.7. Analysis of lncRNA, miRNA, and mRNA Expression

Raw sequencing data were quality-controlled using fastp to remove adapter sequences and low-quality reads, yielding high-quality clean data. Subsequent quantitative analysis was performed with distinct strategies for different RNA types: for mRNA and lncRNA, reads were aligned to the reference genome using HISAT2, and raw read counts were generated with featureCounts; for miRNA, reads were directly aligned to the miRBase database for identification and quantification. Gene expression levels were normalized using TPM (transcripts per million) for consistent visualization and downstream analysis. Differential expression analysis was performed using the DESeq2 R package. Genes with a *p*-value < 0.05 and |log_2_FoldChange| > 1 were defined as significantly differentially expressed [23].

### 2.8. GO and KEGG Enrichment Analysis

Using the Gene Ontology database, we statistically analyzed the number of differentially expressed genes included in each GO term. Concurrently, we performed pathway analysis on the differentially expressed genes using the KEGG database. The significance of gene enrichment in each GO term and pathway entry was calculated using the hypergeometric distribution algorithm. GO terms and KEGG pathway entries with *p*-values < 0.05 were considered significantly enriched.

### 2.9. Construction of lncRNA-miRNA-mRNA Networks

Based on differential mRNA GO enrichment analysis results, genes related to “heart development” were listed as the foundation for subsequent analysis. To construct a high-confidence, resolvable ceRNA regulatory network, lncRNA screening criteria were set at |log2FC| ≥ 4 and *p*-value < 0.05 to ensure statistical significance. The miRanda algorithm was employed to predict target relationships between miRNAs and mRNAs, as well as between miRNAs and lncRNAs. Cytoscape software 3.9.1 was utilized to visualize the lncRNA-based ceRNA network diagram [24,25].

### 2.10. Statistical Analysis

All data are presented as the mean ± standard deviation (SD) from three independent biological replicate experiments (*n* = 3). Statistical analyses were performed using GraphPad Prism 8 software. Prior to intergroup comparisons, the normality and homogeneity of variances of the quantitative data were first verified using the Shapiro–Wilk test and the F-test, respectively. If the data met the assumptions for parametric tests, (un)paired two-tailed Student’s *t*-tests were employed for comparisons. If the assumptions were not met, corrected *t*-tests (e.g., Welch’s *t*-test) were used instead. Any specific statistical methods pertaining to the figures are described separately in their respective captions. For all analyses, a *p* value of < 0.05 was considered statistically significant.

## 3. Results

### 3.1. Sirt1 Inhibits Cardiac Differentiation in hESCs in the Early Stage

**Figure 1:**
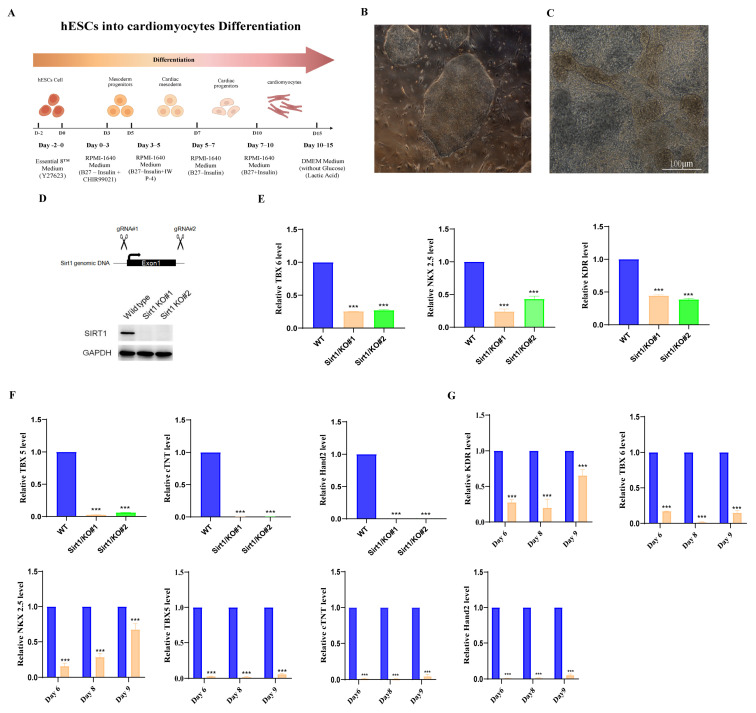
*Sirt1* inhibits cardiac differentiation in hESCs in the early stage. (**A**) Schematic of differentiation of hESC into cardiomyocytes. (**B**) Representative images of embryonic stem cells. (**C**) Morphology of hESC–derived cardiomyocytes, Bar = 100 μm. (**D**) Establishment and validation of *Sirt1* KO hESC lines. (**E**) Detection of expression levels of KDR, TBX6 and NKX2.5 by qRT-PCR. *n* = 3 (*** *p* < 0.001).Blue indicates wild-type group with *Sirt1* not knocked out, yellow indicates *Sirt1* knockout cell line 1, and green indicates *Sirt1* knockout cell line 2; (**F**) Detection of expression levels of TBX5, cTNT and Hand2 by qRT-PCR. *n* = 3 (*** *p* < 0.001).Blue indicates wild-type group with *Sirt1* not knocked out, yellow indicates *Sirt1* knockout cell line 1, and green indicates *Sirt1* knockout cell line 2; (**G**) Detection of functional gene changes in *Sirt1*-knockout hESCs undergoing directed cardiomyocyte differentiation at days 6, 8, and 9 using qPCR. *n* = 3 (*** *p* < 0.001).Blue indicates wild-type group with *Sirt1* not knocked out, yellow indicates *Sirt1* knockout cell line.

A previously validated monolayer culture protocol was employed to induce the differentiation of human embryonic stem cells (hESCs) into cardiomyocytes, yielding efficient cardiomyogenic commitment (Figure 1A,B) [26]. To evaluate the efficacy of this differentiation strategy, morphological alterations were monitored via an inverted microscope (Figure 1C). Subsequently, we employed CRISPR-Cas9 technology to knock out the first exon of *Sirt1* in hESCs, thereby silencing *Sirt1* expression. The expression pattern is shown in Figure 1D. Western blot analysis confirmed the absence of *Sirt1* protein in homozygous KO lines (*Sirt1* KO#1, *Sirt1* KO#2) compared to wild-type (WT) cells (Figure 1D), validating successful gene ablation. Next, we assessed the cardiac differentiation in *Sirt1* KO hESCs. Our results revealed significantly reduced expression of mesodermal markers (TBX6, KDR), cardiomyocyte progenitor markers (NKX2.5, TBX5), and mature cardiomyocyte markers (cTNT, Hand2) in KO cells relative to WT controls (Figure 1E,F). To define the temporal window of *Sirt1* action, we analyzed marker gene expression in *Sirt1* KO cells at days 6, 8, and 9 of differentiation. Our results showed significant downregulation of KDR, TBX6, NKX2.5, TBX5, cTNT, and Hand2 as early as day 6 (Figure 1G). These findings indicate that *Sirt1* is critical for the early stages of hESC cardiac differentiation.

### 3.2. Identification and Analysis of Differentially Expressed mRNAs, miRNAs, and lncRNAs During hESC Cardiomyocyte Differentiation

**Figure 2:**
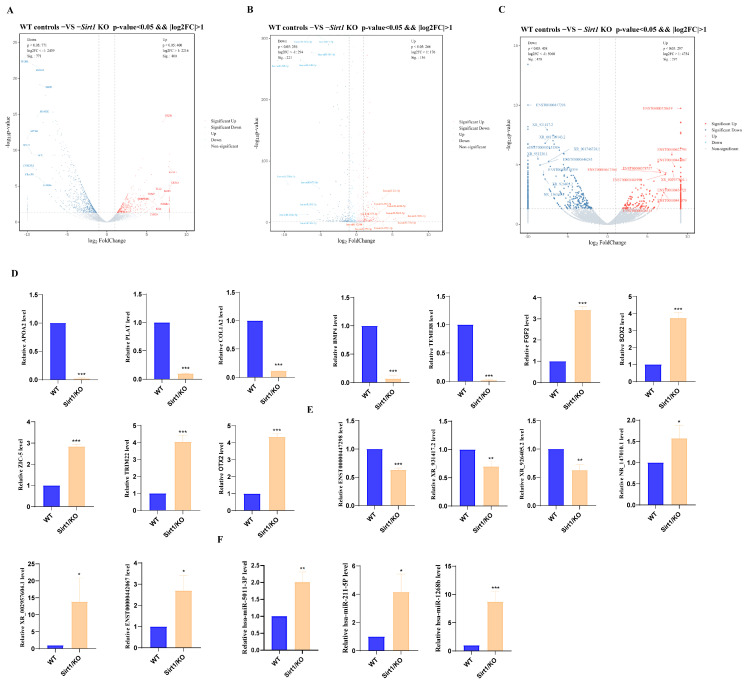
Expression Profiling Analysis of DE mRNAs, miRNAs, and LncRNAs. (**A**) Volcano plot of DE mRNAs. Significantly upregulated (red) and downregulated (blue), *p* < 0.05. (**B**) Volcano plot of DE miRNAs. Significantly upregulated (red) and downregulated (blue), *p* < 0.05, (**C**) Volcano plot of DE LncRNAs. Significantly upregulated (red) and downregulated (blue), *p* < 0.05. (**D**) qPCR validation of DE mRNA expression. *n* = 3 (*** *p* < 0.001). (**E**) qPCR validation of DE lncRNA expression. *n* = 3 (* *p* < 0.05, ** *p* < 0.01, *** *p* < 0.001). (**F**) qPCR validation of DE miRNA expression. *n* = 3 (* *p* < 0.05, ** *p* < 0.01, *** *p* < 0.001).

To identify ncRNAs associated with cardiomyocyte differentiation, we performed whole-transcriptome sequencing on *Sirt1* KO cells and WT controls following 6 days of directed cardiac differentiation. Compared with WT controls, *Sirt1* KO cells exhibited 1171 differentially expressed DE mRNAs (Figure 2A), 316 DE miRNAs (Figure 2B), and 755 DE lncRNAs (Figure 2C). A detailed analysis of selected genes confirmed the expression trends aligned with transcriptomic sequencing outcomes (Figure 2D−F), indicating that the sequencing data are reliable for subsequent analyses.

### 3.3. GO and KEGG Pathway Analysis of Differentially Expressed ncRNAs and mRNAs

**Figure 3:**
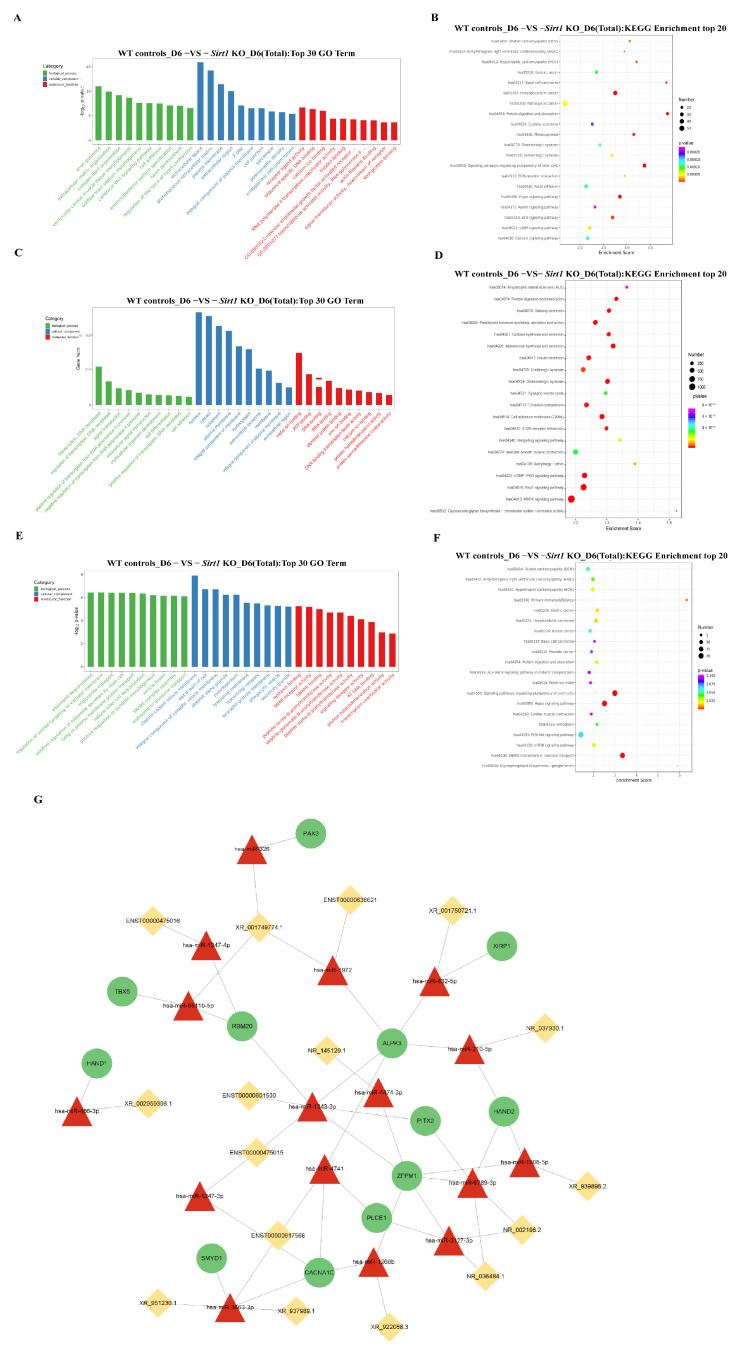
Construction of the ceRNA Regulatory Network and Bioinformatics Analyses. (**A**) GO enrichment analysis of DE mRNAs. (**B**) KEGG pathway analysis of DE mRNAs. (**C**) GO enrichment analysis of DE miRNAs. (**D**) KEGG pathway analysis of DE miRNAs. (**E**) GO enrichment analysis of DE lncRNAs. (**F**) KEGG pathway analysis of DE lncRNAs. (**G**) LncRNA-miRNA-mRNA ceRNA regulatory network diagram.

Based on whole-transcriptome sequencing data, we constructed a ceRNA regulatory network associated with cardiomyocyte differentiation and performed functional enrichment analyses for DE mRNAs, miRNAs, and lncRNAs. GO and KEGG pathway analyses revealed that DE mRNAs were significantly enriched in biological processes and signaling pathways linked to cardiac development, including ventricular cardiomyogenesis, cardiac development, the Hippo signaling pathway, the Wnt signaling pathway, and pluripotency-stemness regulatory pathways (Figure 3A,B). DE miRNAs were primarily involved in processes such as transcriptional regulation and cell differentiation, with significant enrichment in pathways including the cGMP-PKG signaling pathway and cell adhesion molecules (Figure 3C,D). Notably, DE lncRNAs also exhibited marked enrichment in cardiac development-related pathways, such as the Hippo and Wnt signaling pathways (Figure 3E,F).

To further analyze the ceRNA-mediated mechanism by which *Sirt1* promotes cardiomyocyte differentiation, we predicted target relationships of DE ncRNAs using the miRanda algorithm. We screened potential RNA interaction pairs functionally associated with “cardiac development” and ultimately constructed a core ceRNA regulatory network consisting of 15 lncRNAs, 15 miRNAs, and 11 mRNAs (Figure 3G). This network encompassed 44 regulatory pathways, with specific relationships detailed in Table S4. Collectively, these findings suggest that *Sirt1* may influence cardiac development by regulating the post-transcriptional epigenetic landscape mediated by these non-coding RNAs.

### 3.4. Validation of the lncRNA-miRNA-mRNA Axis

**Figure 4:**
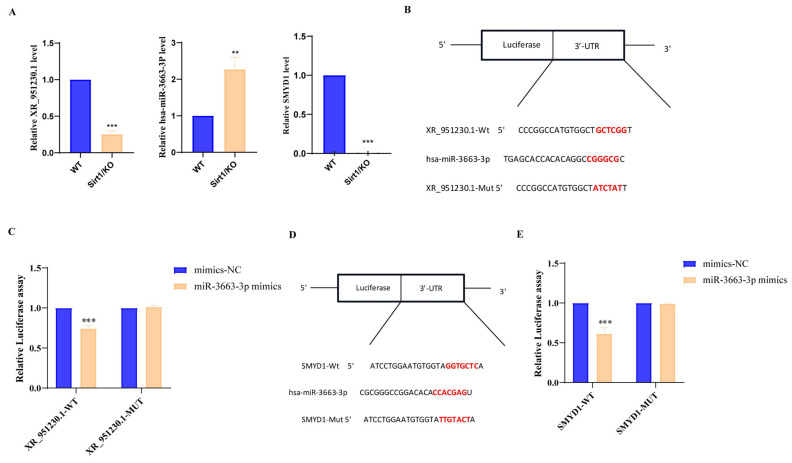
Validation of the XR_951230.1/hsa-miR-3663-3p/SMYD1 Axis. (**A**) qRT-PCR analysis of XR_951230.1, hsa-miR-3663-3p, and SMYD1 expression in WT cells and *Sirt1* KO hESCs, *n* = 3. (** *p* < 0.01, *** *p* < 0.001). (**B**) miRanda-predicted complementary binding sites between XR_951230.1 and hsa-miR-3663-3p. The complementary binding sites are highlighted in red.(**C**) Dual-luciferase reporter assay confirming specific binding between XR_951230.1 and hsa-miR-3663-3p, *n* = 3. (*** *p* < 0.001). (**D**) miRanda-predicted complementary binding sites between hsa-miR-3663-3p and the 3′ UTR of SMYD1 mRNA. The complementary binding sites are highlighted in red. (**E**) Dual-luciferase reporter assay validating specific binding between hsa-miR-3663-3p and the SMYD1 3′ UTR, *n* = 3. (*** *p* < 0.001).

To further confirm the validity of the predicted ceRNA pathway, we selected the XR_951230.1/hsa-miR-3663-3p/SMYD1 axis from the constructed ceRNA network for verification. First, we quantified the expression levels of XR_951230.1, SMYD1, and hsa-miR-3663-3p in *Sirt1* KO cells and WT control hESCs. As shown in Figure 4A, compared to WT cells, XR_951230.1 and SMYD1 expression were significantly downregulated in the *Sirt1* KO cells, whereas hsa-miR-3663-3p expression was markedly upregulated.

Next, we predicted the target-binding relationships among XR_951230.1, hsa-miR-3663-3p, and SMYD1 using the miRanda algorithm and TargetScan database. Computational predictions indicated that XR_951230.1 contains complementary binding sites for hsa-miR-3663-3p (Figure 4B), while hsa-miR-3663-3p can target the 3′ untranslated region (3′ UTR) of SMYD1 mRNA via complementary sequences (Figure 4D). To validate these molecular interactions, we constructed wild-type (WT) and mutant (MUT) dual-luciferase reporter plasmids for the XR_951230.1/hsa-miR-3663-3p and hsa-miR-3663-3p/SMYD1 pairs. Luciferase activity assays showed that co-transfection of WT XR_951230.1 reporter plasmid with hsa-miR-3663-3p mimics significantly reduced luciferase activity, whereas no significant change was observed in the mutant XR_951230.1 group (Figure 4C). Similarly, co-transfection of WT SMYD1 3′ UTR reporter plasmid with hsa-miR-3663-3p mimics led to a significant decrease in luciferase activity, with no alteration in the mutant SMYD1 group (Figure 4E). Collectively, these results demonstrate direct binding between XR_951230.1 and hsa-miR-3663-3p, as well as between hsa-miR-3663-3p and SMYD1, thereby validating the XR_951230.1/hsa-miR-3663-3p/SMYD1 ceRNA axis through which *Sirt1* promotes hESC cardiomyocyte differentiation.

## 4. Discussion

hESCs possess the unique potential to differentiate into cardiomyocytes, providing a powerful in vitro platform for dissecting the intricate molecular circuitry governing cardiac development. Previous studies have demonstrated that *Sirt1* knockout mice exhibit cardiac septal and valvular developmental abnormalities, highlighting a crucial role for *Sirt1* in cardiac morphogenesis [2,27]. However, the specific molecular mechanisms by which *Sirt1* promotes cardiomyocyte differentiation remain incompletely understood.

Cardiac development is a highly coordinated multistep process involving key events such as cell migration, fusion, and lineage-specific differentiation. Transcription factors, including TBX6, TBR, NKX2.5, and TBX3, play central regulatory roles in cardiac lineage determination and differentiation [28,29]. This study systematically investigated the function of *Sirt1* in cardiomyocyte differentiation by establishing a *Sirt1* knockout human embryonic stem cell (hESC) model. Results demonstrated that *Sirt1* deficiency significantly impeded the directed differentiation of hESCs into cardiomyocytes, leading to marked downregulation of key genes, including TBX6, TBR, NKX2.5, TBX5, cTNT, and Hand2. Further analysis revealed that *Sirt1* depletion primarily affected the early stages of cardiomyocyte differentiation. Furthermore, prior studies indicate that in embryonic stem cell differentiation systems, *Sirt1* specifically binds CRABPII and regulates its acetylation status, thereby influencing the nuclear–cytoplasmic distribution of retinoic acid (RA). *SIRT1* deficiency leads to excessive CRABPII acetylation and nuclear accumulation, thereby enhancing RA signaling and ultimately causing multiple developmental defects in mice [30]. Based on this mechanism, we hypothesize that *Sirt1* may regulate directed cardiomyocyte differentiation in human embryonic stem cells in vitro in a similar way. Collectively, these findings indicate that Sirt1, as a key regulatory factor, plays a central role in initiating and driving cardiomyocyte differentiation.

ceRNA regulatory networks have emerged as critical modulators of cardiac development and cardiomyocyte differentiation. For instance, Sun R et al. discovered that LncRNA MALAT1 functions as a ceRNA to sequester miR-200a-3p, thereby upregulating PDCD4 expression and inhibiting hypoxia-induced cardiomyocyte apoptosis [31]. Similarly, Chen G et al. reported that lncRNA CRRL regulates the target gene Hopx by competitively binding miR-199a-3p, thereby influencing cardiomyocyte regeneration and cardiac repair [32]. Based on whole-transcriptome sequencing data, we identified DE non-coding RNAs with mRNAs associated with “cardiac development,” and we constructed a ceRNA regulatory network. Using dual-luciferase reporter assays, we validated the existence of the XR_951230.1/hsa-miR-3663-3p/SMYD1 ceRNA axis. Furthermore, we found that *Sirt1* knockout downregulates XR_951230.1 and SMYD1 expression while upregulating hsa-miR-3663-3p levels, suggesting a *Sirt1*-dependent regulatory mechanism for this ceRNA axis.

SMYD1 (also known as BOP), a striated muscle-specific histone methyltransferase, plays an indispensable role in cardiac development. Studies have shown that SMYD1 deficiency leads to embryonic lethality in mice, accompanied by severe right ventricular hypoplasia [33,34]. Additionally, SMYD1 regulates cardiac energy metabolism by targeting genes such as Perm1 [35]. In our study, *Sirt1* sustains XR_951230.1 expression, which functions as a molecular sponge for hsa-miR-3663-3p to relieve SMYD1 from suppression and facilitate cardiomyocyte differentiation. *Sirt1* depletion reduces XR_951230.1 levels, impairing its hsa-miR-3663-3p sequestration and subsequent SMYD1 expression. This disrupts downstream mesodermal and cardiomyocyte gene cascade activation, inhibiting normal cardiomyocyte differentiation.

However, this study has several limitations that need to be acknowledged. Although we have proposed a potential ceRNA regulatory axis—“XR_951230.1/miR-3663-3p/SMYD1”—based on transcriptome sequencing and dual-luciferase reporter assays, its causality and completeness still require further validation. Specifically, functional rescue experiments are needed to determine whether such interventions can reverse the differentiation defects, thereby providing direct causal evidence for this pathway. Secondly, the upstream mechanism by which *Sirt1* promotes the expression of XR_951230.1 remains unclear. Follow-up studies should employ techniques such as ChIP-qPCR to verify whether *Sirt1* directly binds to the promoter region of this long non-coding RNA and regulates its transcription. Moreover, all conclusions are derived from a two-dimensional in vitro model; the physiological role of this axis in cardiac development and homeostasis ultimately needs to be validated using in vivo models. Additionally, more precise and systematic functional measurements based on larger-scale experiments are required, such as quantitatively assessing the population of cTnT^+^ cells via flow cytometry, and thoroughly evaluating cardiomyocyte functional maturity through contractility analysis and calcium handling assays.

In summary, this study establishes *Sirt1* as a critical regulator of directed hESC differentiation into cardiomyocytes and reveals a novel mechanism whereby *Sirt1* mediates this process via the XR_951230.1/miR-3663-3p/SMYD1 ceRNA axis. These findings provide new insights into the molecular regulatory networks governing cardiac development and may identify potential therapeutic targets for stem cell-based cardiac disease therapy.

## Data Availability

The original contributions presented in this study are included in the article/Supplementary Materials.

## Supplementary Materials

The following supporting information can be downloaded at https://www.mdpi.com/article/10.3390/genes17030282/s1, Table S1: *Sirt1* gRNA Primer Sequence List; Table S2: Sequences of the wild-type (WT) and mutant (Mut) inserts in the dual-luciferase reporter plasmids; Table S3: Primer Sequences; Table S4: Predicted LncRNAs-miRNAs-mRNAs Relationship Pairs.

## Abbreviations

The following abbreviations are used in this manuscript:

DE ncRNAs	Differentially Expressed ncRNAs
ncRNAs	Non-coding RNAs
RT-qPC	Real-Time QuantitativePCR
KEGG	Kyoto Encyclopedia of Genes and Genomes
GO	Gene Ontology
